# Don’t be scared to touch! Effectiveness of a new disinfection technology based on Ag ions & Zeolite: Valerio Peruzzi

**DOI:** 10.1093/eurpub/ckac131.078

**Published:** 2022-10-25

**Authors:** V Peruzzi, D Amodeo, I De Palma, G Messina

**Affiliations:** 1 Post Graduate School of Public Health, University of Siena, Siena, Italy; 2 Department of Medical Biotechnologies, University of Siena, Siena, Italy; 3 Department of Molecular and Developmental Medicine, University of Siena, Siena, Italy

## Abstract

**Background:**

Disinfection of contact surfaces has become common practice since the two-year Covid-19 pandemic. The transmission of microbial agents has long been the focus of public health and hygiene awareness campaigns. Indeed, the development of new disinfection technologies and approaches is attracting considerable interest in the scientific community. Mixed plastic powders with antimicrobial properties and silver ions that compromise the metabolism of microorganisms could reduce the contamination of the contact surfaces. We aimed to evaluate an inorganic antimicrobial agent (IAA) based on Ag ions and zeolite mixed in a resin.

**Methods:**

This experimental study was carried out at the University of Siena, Italy. Different objects were produced in two versions: i) with an IAA mixed in plastic resin; ii) with a standard plastic mixture. To the eye, the two versions were indistinguishable and were randomly contaminated with the hands of several operators. After the hand contamination, T0, we sampled the objects using RODAC plates at T1 (1h) and T2 (6h), incubating at 36 °C for 48 hours. Comparisons of the biocidal effect were made at T1 and T2. Statistical analysis was carried out with Stata.

**Results:**

The mean level of contamination of the objects made with standard plastic were, respectively 50 CFU (SD 36.5) at T1 and 20 CFU (SD 13.6) at T2. In comparison, the objects made with IAA resin showed a mean level of contamination of 10 CFU (SD 5.9) at T1 and 6 CFU (SD3.6) at T2. The objects made with IAA resin have shown a mean percentage reduction of contamination of 79.5% at T1 and 78.3% at T2.

**Conclusions:**

IAA resin reduced contamination on objects that came into contact with hands. Antimicrobial plastic blends, are a valuable aid in counteracting the spread of infection related to contact with surfaces and fomites. The public health system could support and raise awareness for using these innovative materials for everyday applications and in healthcare facilities.

**Key messages:**

• Inorganic antimicrobial agent based on Ag ions and Zeolite mixed in a resin are efficient in reducing the contamination on different items in a real-life context.

• Public health system have to support and sensitize to production with inorganic materials with proven antimicrobial properties.

